# Patching the gaps towards the 90–90–90 targets: outcomes of Nigerian children receiving antiretroviral treatment who are co-infected with tuberculosis

**DOI:** 10.7448/IAS.18.7.20251

**Published:** 2015-12-02

**Authors:** Dick D Chamla, Chukwuemeka Asadu, Abiola Davies, Arjan de Wagt, Oluwafunke Ilesanmi, Daniel Adeyinka, Ebun Adejuyigbe

**Affiliations:** 1 Health Section, UNICEF, New York, NY, USA; 2 Federal Ministry of Health, NASCP, Abuja, Nigeria; 3 Country Office, UNICEF, Abuja, Nigeria; 4 World Health Organization, Abuja, Nigeria; 5 Obafemi Awolowo University, Ile-Ife, Nigeria

**Keywords:** children, tuberculosis, HIV, ART

## Abstract

**Introduction:**

Nigeria has a high burden of children living with HIV and tuberculosis (TB). This article examines the magnitude of TB among children receiving antiretroviral treatment (ART), compares their ART outcomes with their non-TB counterparts and argues that addressing TB among children on ART is critical for achieving the 90–90–90 targets.

**Methods:**

This was a facility-based, retrospective analysis of medical records of children aged <15 years who were newly initiated on ART between 2011 and 2012. Structured tools were used to collect data. STATA software was used to perform descriptive, survival and multivariate analyses.

**Results:**

A total of 1142 children with a median age of 3.5 years from 20 selected facilities were followed for 24 months. Of these, 95.8% were assessed for TB at ART initiation and 14.7% had TB. Children on ART were more likely to have TB if they were aged 5 years or older (*p<*0.01) and had delayed ART initiation (*p<*0.05). The cotrimoxazole and isoniazid prophylaxes were provided to 87.9 and 0.8% of children, respectively. The rate of new TB cases was 3 (2.2–4.0) per 100 person-years at six months and declined to 0.2 (0.06–1.4) per 100 person-years at 24 months. TB infection [adjusted hazard ratio (aHR): 4.3; 2.3–7.9], malnutrition (aHR: 5.1; 2.6–9.8), delayed ART initiation (aHR: 3.2; 1.5–6.7) and age less than 1 year at ART initiation (aHR: 4.0; 1.4–12.0) were associated with death. Additionally, patients with TB (aHR: 1.3; 1.1–1.6) and children below the age of 1 at ART initiation (aHR: 2.9; 1.7–5.2) were more likely to be lost to follow-up (LFU).

**Conclusions:**

Children on ART with TB are less likely to survive and more likely to be LFU. These risks, along with low isoniazid uptake and delayed ART initiation, present a serious challenge to achieving the 90–90–90 targets and underscore an urgent need for inclusion of childhood TB/HIV in global plans and reporting mechanisms.

## Introduction

Tuberculosis (TB) is common in countries that have a high burden of children living with HIV and high under-five mortality [1]. Yet, TB/HIV co-morbidity among children has been largely overlooked globally and grossly under-reported. In most countries, the proportion of children with TB/HIV co-morbidity remains unknown. The most recent global WHO TB report could not disaggregate data on the burden or trends of TB/HIV among children [2]. Despite advances in early infant diagnosis of HIV infections, there are still challenges in diagnosing TB in children [3]. These diagnostic challenges are further exacerbated in children with HIV because clinical and radiological manifestations of TB are modified [4]. Additionally, opportunities for identifying HIV-infected children in child survival programs, including TB clinics, continue to be missed [5]. TB co-treatment in children with HIV presents a significant challenge for children aged less than three years, due to interaction of rifampicin with lopinavir/ritonavir and nevirapine [6,7]. Despite these obstacles, there is reason for optimism in tackling childhood TB/HIV supported by the presence of the global Roadmap for Childhood Tuberculosis with a goal of zero TB deaths [8], a Double Dividend initiative with the dual goal of improving paediatric HIV and child survival rates [9] and an ambitious post-2015 WHO TB strategy [10] that attempts to engage broader maternal and child health communities.

In Nigeria, the proportion of notified TB cases with HIV-positive test results was 22% in 2013 [2], although large variations among states have been reported [11]. High mortality among populations with TB/HIV has been well documented [12,13]. The coverage of paediatric antiretroviral treatment (ART) remains low [14], while high lost to follow-up (LFU) rates among children on ART has also been reported [15]. With high rates of mother-to-child transmission of HIV [14], the number of children living with HIV and in need of ART will continue to rise, yet data on the burden and outcomes of TB-infected children receiving ART remain limited. This article aims to examine the magnitude of TB among children starting ART and to compare their ART outcomes with non-TB-infected children. We argue that addressing TB is critical for accelerating progress towards the new global targets of 90–90–90 for the diagnosis, treatment and viral suppression needed to improve survival of children living with HIV.

## Methods

This was a facility-based, retrospective analysis of medical records of a cohort of children from selected health facilities who were newly initiated on ART between 1 January 2011 and 31 December 2012. This analysis was conducted as part of larger paediatric HIV assessment supported by United Nations Children Fund and the World Health Organization, in collaboration with the Nigeria Federal Ministry of Health and members of academic institutions and partners from the national paediatric ART technical working group. The eligibility criteria were strictly limited to children aged <15 years who had initiated ART between 1 January 2011 and 31 December 2012. The maximum follow-up period was 24 months after ART initiation. At the time of the assessment, Nigeria ART guidelines were based on the 2010 WHO guidelines that recommended early infant diagnosis at six weeks for all HIV-exposed infants and initiation of ART for all HIV-infected children under the age of 2 years, at CD4 count thresholds of ≤750 cells/mm^3^ or %CD4+≤25 for children aged 24 to 59 months and ≤350 cells/mm^3^ for children aged 5 years and older [16].

### TB diagnosis

TB screening and diagnosis in facilities followed the revised *National Tuberculosis and Leprosy Control Programme Workers’ Manual* (5th edition). A symptomatic checklist established TB suspects using the following symptoms: cough for two weeks or more; unexplained weight loss; failure to thrive and/or malnutrition; and history of contact with a TB case. A TB diagnosis was determined by a clinician based on sputum results for children who produced sputum radiological examination or culture. The study only collected data on TB assessment and confirmed TB diagnoses as recorded in ART registers. Data on TB suspects and diagnostic methods used were not routinely recorded in ART registers and were not included in the analysis.

### Sampling and data sources

Purposive sampling was used to select a total of 20 public and private facilities offering paediatric ART services from five states (Anambra, Bayelsa, Benue, Kano and Lagos). These represented a mixture of high and low HIV burden in five geopolitical zones. The list of facilities were drawn from the Nigerian health facility directory and sorted by key domains for wide representation. These included urban/rural location and ownership of facility (public/private/faith-based). Facility level was not included as a criterion, as only tertiary and general hospitals formally initiate paediatric ART in Nigeria. All eligible children who had newly initiated ART between January 2011 and December 2012 were selected for analysis.

The primary sources of paediatric ART data were paper-based or electronic HIV care/ART patients’ cards, patient charts and ART registers that contained data elements for TB assessments and diagnoses. Patient-level medical records were extracted into a structured data collection tool in English. Collected data included demographic characteristics, clinical and laboratory reports, TB status, uptake of isoniazid (INH) and cotrimoxazole, ART regimens used and ART outcomes.

### Outcome measures

The primary outcome measures were retention, LFU and death recorded at 6, 12 and 24 months following initiation of ART, comparing patients on ART with TB to those without TB at baseline. The definition of *LFU* followed the criteria articulated in the national ART guidelines, which is missed clinic appointments or pharmacy antiretroviral (ARV) refills for 90 days following last scheduled appointment as shown in the patient's records. Secondary outcome measures included new TB occurrence during HIV treatment at 6, 12 and 24 months after initiation of ART and uptake of INH and cotrimoxazole.

Individual level factors that often influence retention, LFU and deaths based on various literature were selected for analysis [17]. CD4 percentage or counts were measured at ART initiation, 6, 12 and 24 months to determine the degree of immunosuppression. Based on the 2010 WHO guidelines, *severe immunosuppression* was defined as an initial CD4 count less than 750 cells/mm^3^ or a percentage less than 15% for patients less than two years of age, less than 500 cells/mm^3^ or a percentage less than 15% for patients between two and five years of age and less than 200 cells/mm^3^ or a percentage less than 15% for patients five years or older. Other categories of immunosuppression, *moderate* and *no immunosuppression* as articulated in the WHO guidelines, were also analyzed. For malnutrition we calculated weight-for-age *z*-scores and categorized children as *z*-score less than or greater than −2 standard deviation [18].

Time to ART initiation was estimated by subtracting the dates of HIV diagnosis from ART initiation. We followed Kim *et al*.'s definition of *prompt initiation of ART* as those children initiating ART within 21 days [19]. Other individual factors included for analysis were age, referral sources (entry points) to ART, age and ARV regimens at ART initiation, sex and facility ownership (public/private).

### Data analysis

Kaplan–Meier survival and Nelson–Aalen cumulative hazard analyses were used to estimate survival and LFU probabilities, respectively, through 24 months after ART initiations comparing children on ART who had TB and non-TB counterparts. Bivariate and multivariate Cox proportion hazard models were used to establish the relationships between the individual factors mentioned above and primary outcome measures. Using the survival analysis function, the rates and their corresponding 95% confidence intervals (CI) of new TB cases, LFU and deaths were estimated and expressed per 100 person-years. The statistical associations between dependent and independent variables were estimated using hazard ratios (HR) for survival analysis, while odds ratios (OR) with 95% CI were used to establish correlates of TB at ART initiation. The proportional hazards assumption were assessed by graphical methods and Harrell's C measure of concordance probability. Statistical significance was determined at *α<*0.05. All statistical analyses were completed using STATA version IC 11 (StataCorp LP, College Station, TX, USA).

### Ethical considerations

Ethical clearance for the study was granted by National Health Research Ethics Committee of Nigeria (NHREC). Formal requests and approvals to visit facilities and abstract data were also granted by the Federal and State Ministries of Health. Confidentiality of patient records was assured by the use of unique number allocation and removal of personal identifiers.

## Results

The records from 1142 children aged less than 15 years who were newly initiated on ART between January 2011 and December 2012 were analyzed from 20 selected facilities. The majority of records, 984 (86.4%), were from public/government facilities, and less than 1.1% were from private for-profit facilities. Most (62.6%) initiated ART at tertiary facilities, with a median age (at ART initiation) of 3.5 years [interquartile ratio (IQR): 1.6–7.2] and 618 (54.7%) being males. Approximately 16.4% of those initiating ART were less than one year of age, 44.9% were between one and five years of age, 25.4% were aged between five and ten years and 13.2% were aged 10 years and above. At baseline, 50.1% (507/1002) of children with CD4 results were severely immunocompromised and 12.1% were malnourished. The most-used regimen at ART initiation was AZT/3TC/NVP (75.5%) followed by AZT/3TC/EFV (13.1%). Only 1.1% of patients were initiated with ritonavir-boosted lopinavir-based regimens.

### TB burden

The TB status was assessed and determined in 1094 (95.8%) patients. Of these, 161 (14.7%) were diagnosed as having active TB. In multivariate regression adjusting for other factors, children on ART were more likely to be diagnosed with TB if they were five years old or above at ART initiation (*p<*0.01) and had delayed ART initiation (*p<*0.05). There was no significant statistical difference in severe immunosuppression between children with and without TB in the final multivariate regression model (*p>*0.05) despite its independent association with TB following bivariate analysis (Table 1).

**Table 1:** Correlates of TB among children on ART

Factor	Co-infected with TB	No TB	OR (95% CI)	*p*	aOR (95% CI)	*p*
Age at ART initiation (*n*=1094)	161	933				
<1 year (reference)	16 (9.9%)	154 (16.5%)				
2 to <5 years	57 (35.4%)	419 (44.9%)	1.43 (0.78–2.60)	0.25	1.74 (0.65–1.81)	0.28
5 to 9 years	63 (39.1%)	237 (25.4%)	3.18 (1.75–5.81)	0.001	4.41 (1.61–12.07)	0.004
10 to 14 years	25 (15.5%)	123 (13.2%)	2.36 (1.18–4.72)	0.02	3.53 (1.20–10.35)	0.022
Sex (*n*=1081)	160	921				
Female (reference)	69 (43.1%)	414 (45%)				
Male	91 (56.9%)	507 (55%)	1.08 (0.77–1.51)	0.67		
Facility ownership (*n*=1091)	160	931				
Public/government (reference)	148 (92.5%)	810 (87%)				
Private not for profit	11 (6.9%)	110 (11.8%)	0.55 (0.29–1.04)	0.07		
Private for profit	1 (0.6%)	11 (1.2%)	0.49 (0.06–3.88)	0.51		
CD4 at ART initiation (*n*=1002)	131	871				
Severe immunosuppression	76 (58%)	431 (49.5%)	3.49 (1.74–6.79)	0.001	1.70 (0.80–3.59)	0.16
Nutrition status (*n*=988)	160	828				
Normal (reference)	137 (85.6%)	731 (88.3%)				
Malnourished	23 (14.4%)	97 (11.7%)	1.31 (0.79–2.19)	0.29		
Time to ART initiation (*n*=1052)	156	896				
Prompt initiation (reference)	19 (12.2%)	256 (28.6%)				
Delayed initiation	137 (87.8%)	640 (71.4%)	2.88 (1.75–4.76)	0.001	2.38 (1.23–4.62)	0.01
Referral sources to ART initiation (*n*=1094)	161	933				
HIV counselling and testing clinic (reference)	127 (78.9%)	633 (67.8%)				
Paediatric outpatient clinic	29 (18%)	207 (22.2%)	0.69 (0.45–1.08)	0.10	1.08 (0.65–1.81)	0.76
PMTCT clinic	1 (0.6%)	52 (5.6%)	0.10 (0.01–0.70)	0.02	0.28 (0.04–2.18)	0.23
Others	4 (2.5%)	41 (4.4%)				
ARV regimens at ART initiation (*n*=1092)	160	932				
AZT/3TC/NVP	30 (18.8%)	793 (85.1%)				
AZT/3TC/EFV	91 (56.9%)	52 (5.6%)				
AZT/3TC/ABC (triple nuke)	31 (19.4%)	10 (1.1%)				
D4T/3TC/NVP	2 (1.3%)	45 (4.8%)				
Others	6 (3.8%)	32 (3.4%)				

TB, tuberculosis; ART, antiretroviral therapy; OR, odds ratio; aOR, adjusted OR; CI, confidence interval; PMTCT, prevention of mother-to-child transfer; ARV, antiretroviral.

The main ART regimen for children with TB was AZT/3TC/EFV (56.9%) followed by AZT/3TC/ABC (19.4%) and AZT/3TC/NVP (18.8%). The cotrimoxazole prophylaxis was provided to 87.9% at ART initiation, including 95.7% (154/161) of those with TB. The median age at cotrimoxazole initiation was 4.7 years (IQR: 1.6–7.8) and 2.8 years (IQR: 1.3–6.4) for those with and without TB, respectively. Only 0.8% (7/933) of non-TB children were reported as receiving INH prophylaxis.

At the 24 month follow-up, 7.3% (68/933) of non-TB children developed TB, with 50 (73.5%) of these new TB cases occurring in the first six months. The rate of new TB cases was 3 per 100 person-years (95% CI: 2.2–4.0) at six months which declined to 0.9 (0.5–1.5) and 0.2 (0.06–1.4) per 100 person-years at 12 and 24 months, respectively.

### Retention, LFU and deaths among TB co-infected and non-infected

The differences in retention, LFU and deaths at the 6, 12 and 24 month follow-up periods among children on ART with and without TB is shown in Table 2. Both mortality (*p<*0.01) and LFU (*p<*0.05) were higher among children on ART who had been diagnosed with TB compared to non-TB children. The rates of LFU and death at 24 months among TB co-infected children were 12.8 (95% CI: 8.8–18.6) and 8 (5.1–12.8) compared to 8.7 (7.3–10.6) and 2.3 (1.6–3.2) per 100 person-years for non-TB children.

**Table 2:** Outcomes among children on ART by TB status

	Children with TB	Non-TB children	OR (95% CI)	*p*
6 months after ART initiation	161	933		
Retained (reference)	123 (76.4%)	844 (90.5%)		
Died	18 (11.2%)	22 (2.4%)	5.6 (2.9–10.6)	0.00
Lost to follow-up	20 (12.4%)	67 (7.2%)	2.1 (1.2–3.5)	0.01
12 months after ART initiation^a^	160	932		
Retained (reference)	114 (71.3%)	803 (86.1%)		
Died	19 (11.9%)	24 (2.6%)	5.2 (2.7–9.9)	0.00
Lost to follow-up	27 (16.9%)	105 (11.3%)	1.8 (1.1–2.9)	0.01
24 months after ART initiation^a^	129	805		
Retained (reference)	78 (60.5%)	615 (76.4%)		
Died	18 (14.0%)	35 (4.3%)	4.2 (2.3–7.8)	0.00
Lost to follow-up	33 (25.5%)	155 (19.3%)	1.7 (1.1–2.6)	0.03

TB, tuberculosis; ART, antiretroviral therapy; OR, odds ratio.

a Data analysis excludes participants who transferred out.

**Figure 1:**
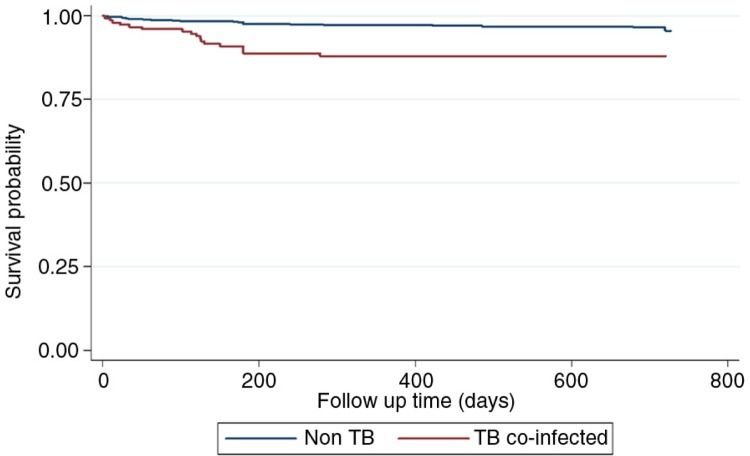
Kaplan–Meier survival by tuberculosis status among children on antiretroviral therapy.

**Figure 2:**
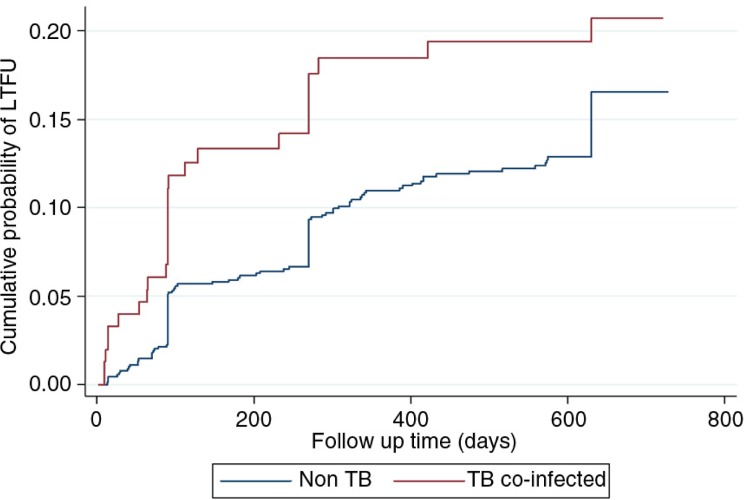
Nelson–Aalen loss to follow-up by tuberculosis status.

Following the Kaplan–Meier survival and Nelson–Aalen cumulative hazard analyses, children on ART who had TB were less likely to survive (*p=*0.001) and at increased risk to being LFU compared to those without TB (Figures 1 and 2). In a multivariate Cox proportional hazards model (Table 3), children with TB (aHR: 4.3; 95% CI: 2.3–7.9), malnutrition (aHR: 5.1; 95% CI: 2.6–9.8), those who delayed ART initiation (aHR: 3.2; 95% CI: 1.5–6.7) and those who were less than one-year old at ART initiation (aHR: 4.0; 95% CI: 1.4–12.0) were more likely to die. Additionally, children less than one year of age at ART initiation (aHR: 2.9; 95% CI: 1.7–5.2) and those with TB (aHR: 1.3; 95% CI: 1.1–1.6) were more likely to be LFU.

**Table 3:** Factors associated with mortality and loss to follow-up

	Mortality				Loss to follow-up			
		
	HR (95% CI)	*p*	aHR (95% CI)	*p*	HR (95% CI)	*p*	aHR (95% CI)	*p*
Age at ART initiation								
<1 year	6.6 (2.4–17.7)	0.001	6.4 (1.9–12.0)	0.002	2.8 (1.6–4.9)	0.0001	3.2 (1.6–6.3)	0.001
2 to <5 years	2.9 (1.1–7.9)	0.03	2.8 (1.0–7.5)	0.04	1.8 (1.07–2.9)	0.025	1.8 (1.0–3.3)	0.05
5 to 9 years (reference)								
10 to 14 years	2.5 (0.8–8.3)	0.13 (NS)	1.8 (0.5–6.8)	0.37	1.7 (0.9–3.3)	0.098	1.5 (0.7–3.4)	0.31
Sex								
Female (reference)								
Male	1.4 (0.8–2.3)	0.26 (NS)			1.0 (0.8–1.4)	0.82		
Facility ownership								
Public/government (reference)								
Private not for profit	0.6 (0.3–1.6)	0.33 (NS)			0.9 (0.9–1.5)	0.85		
Private for profit	1.8 (0.2–12.8)							
CD4 at ART initiation								
Severe immunosuppression by age	1.3 (0.5–3.2)	0.58 (NS)			1.1 (0.7–1.8)	0.72		
Time to ART initiation								
Prompt initiation (reference)								
Delayed initiation	5.1 (2.1–9.1)	0.026	3.2 (1.5–6.7)	0.002	6.4 (4.6–8.8)	0.007	1.7 (0.4–1.3)	0.39
TB	3.4 (1.9–6.1)	0.0001	6.4 (3.3–12.4)	0.001	1.3 (1.06–1.5)	0.009	2.5 (1.5–4.2)	0.001
Nutrition status								
Normal (reference)								
Malnourished	6.8 (6.8–12.0)	0.0001	5.1 (2.6–9.8)	0.001	1.6 (0.9–2.6)	0.09		
Referral sources to ART initiation								
HIV counselling and testing clinic (reference)								
Paediatric outpatient clinic	1.3 (0.7–2.5)	0.32 (NS)			1.7 (1.2–2.4)	0.002	1.3 (0.7–2.2)	0.39
PMTCT clinic	0.4 (0.1–2.7)	0.33 (NS)			0.9 (0.4–2.0)	0.87 (NS)	0.6 (0.4–3.9)	0.84

ART, antiretroviral therapy; HR, hazard ratio; aHR, adjusted HR; CI, confidence interval; OR, odds ratio; TB, tuberculosis; PMTCT, prevention of mother-to-child transmission.

## Discussion

Few studies have reported the burden and outcomes of children on ART who had TB, and this analysis provides critical evidence that children on ART with TB are more likely to die and be lost to follow up than their non-TB counterparts. This result is consistent with other findings [13,20]. The study further reaffirms published literature of the association of mortality during ART with younger age at ART initiation and malnutrition [17,21].

The high TB screening rate among children on ART in this study is supported by previous studies in Nigeria [15]. The finding that TB increased with age is likely due to late initiation of ART depicted in this study and is similar with other findings in the Africa region [22,23]. This finding may reflect the difficulties in diagnosing TB in younger age groups due to limitations in the utility and interpretation of widely used diagnostic approaches such as chest radiography, sputum microbiology and tuberculin test in HIV/TB co-infected children [24–26]. In contrast, our study did not find significant differences in severe immunosuppression between TB-co-infected and non-infected children. The association between low CD4 count and TB among adult ART patients has also shown mixed results, with some studies showing stronger association and others weaker [22,27].

Higher incident TB infections, particularly within the first six months after ART initiation, is consistent with findings among adult populations where high probability of TB infection in the first three months of ART initiation was discussed [22,27]. Significant differences have been reported between TB before and after ART initiation with the probability of having TB within 30 months for pre-ART individuals as high as 22% compared to 18% among the ART population [27]. The reduction of TB risk following ART has been confirmed in most settings [28] and underscores the need for increased ART coverage and early ART initiation.

The choice of which ARV regimen to use among children and its challenges, particularly in TB/HIV co-infected children, has been amply discussed [7]. Over 18% of children with TB in this study were initiated on a nevirapine-based regimen, which has been found to interact with the TB agent rifampicin [6]. Though anti-TB regimens used were not examined, the Nigeria national guidelines recommend a rifampicin-based regimen as the first line for paediatric TB. Data on switching regimens or adverse reactions to determine any negative consequences for their co-administration were beyond the scope of this paper. These results will likely inform the selection and the optimal list of ARV formulations for paediatric TB/HIV co-morbidity.

The finding of high uptake of cotrimoxazole among children on ART in Nigeria is similar with other research [15], reflecting a concordance to the WHO guidelines and Stop TB policy on TB/HIV collaboration [29]. The significant gap was in the uptake of INH prophylaxis. This corresponds to other African countries [2] and needs urgent attention as improved outcomes have been documented following INH prophylaxis among individuals on ART [30,31]. There are various studies that have proposed measures for improving uptake of and adherence to INH [32–37]. Some of these strategies can be adapted in the Nigerian setting and will likely have a wider impact if implemented in lower-level facilities. The impact of decentralization on the scale-up of services including paediatric ART services has already be ascertained [38] and could well be adapted in Nigeria to increase the uptake of INH prophylaxis.

In 2014, UNAIDS and the international community endorsed new global targets (90–90–90) that seek to ensure that 90% of children living with HIV know their HIV status, 90% of children infected with HIV are receiving ART and 90% of those on treatment are virally suppressed [39]. From the findings of this study, these targets will not be actualized if TB among children living with HIV continues to be excluded from global priorities. Higher rates of mortality and LFU among TB co-infected children, as identified by this study, are detrimental to the progress towards these targets. Other studies have also established an independent association between TB and virological failure, particularly in children co-treated with protease inhibitors [40].

TB and delayed ART initiation have been previously found to be associated with poorer virological response and increased mortality [40–42], supporting the relationship between TB, delayed ART initiation and higher mortality observed in this study. Fewer infants initiated ART in this study, and fewer children were referred from prevention of mother-to-child transfer, which may explain the delay in ART initiation. This highlights the need for improved linkages between entry points for HIV testing and paediatric ART, as well as the adaptation of recent WHO ART guidelines that recommend HIV treatment for all children under the age of 5 and higher CD4 eligibility [43] in order to promote early initiation of ART.

Malnutrition was also identified as a factor associated with mortality. Other studies have established the association between food insecurity and incomplete viral suppression among children on ART [44], emphasizing the importance of improving nutrition among children on ART for survival and progress towards the 90–90–90 targets. Viral load testing, though not a major part of national recommendation at the time of this study, is critical as it is one of the major requirements for tracking progress towards the 90–90–90 targets. The data further underscore the need for improved retention and additional focus on younger age groups with the highest mortality.

The results of this study are limited by several factors. Random selection of facilities was not done in order to ensure that a wide and diverse representation of facilities were included. Data gaps due to incomplete medical records and poor linkage between ART and TB registers were common, though efforts were made to fill in data gaps from alternative sources of data in the facilities, such as clinical charts or laboratory registers. Due to diagnostic challenges, TB diagnosis was likely underestimated in some facilities. Despite these limitations, the findings have been consistent with other reports and have provided preliminary proof of high burden and poorer outcomes for children on ART with TB compared to non-TB children on ART, which may hamper progress towards the 90–90–90 targets.

## Conclusions

TB among children on ART presents a major challenge to achieving the global targets of 90–90–90 requiring global attention. Despite diagnostic challenges, Nigeria has demonstrated that childhood TB screening is effective in identifying prevalent and incident TB cases at different intervals of HIV treatment. However, uptake of INH prophylaxis continues to remain low. Higher levels of mortality, higher rates of LFU and delayed initiation of ART among children with TB need to be addressed to assist progress towards the 90–90–90 targets. These findings underscore the urgent need for inclusion of TB/HIV co-morbidity among children in global plans and reporting mechanisms.
